# Smoking Cessation and Reduction in Schizophrenia (SCARIS) with e-cigarette: study protocol for a randomized control trial

**DOI:** 10.1186/1745-6215-15-88

**Published:** 2014-03-22

**Authors:** Pasquale Caponnetto, Riccardo Polosa, Roberta Auditore, Giuseppe Minutolo, Maria Signorelli, Marilena Maglia, Angela Alamo, Filippo Palermo, Eugenio Aguglia

**Affiliations:** 1 Smoking Prevention/Cessation Centre, A.O.U, Policlinico-V.Emanuele, Department of Clinical and Molecular Biomedicine, University of Catania, Catania, Italy; 2 Institute of Internal Medicine, AziendaOspedaliero-Universitaria “Policlinico-V.Emanuele”, Department of Clinical and Molecular Biomedicine, Università di Catania, Catania, Italy; 3 CTA-Villa Chiara Psychiatric Rehabilitation Clinic and Research, Mascalucia, Catania 95030, Italy; 4 U.O.P.I. of Psychiatry, Department of Clinical and Molecular Biomedicine, Policlinico-Vittorio Emanuele Hospital, University of Catania, Via Santa Sofia 78, Catania 95100, Italy; 5 Department of Clinical and Molecular Biomedicine, A. O. “Garibaldi-Nesima”, University of Catania, Catania, Italy

**Keywords:** Smoking cessation, Smoking reduction, Electronic cigarette, Schizophrenia clinical trial, Cognitive, Addiction, Quality of life

## Abstract

**Background:**

It is well established in studies across several countries that tobacco smoking is more prevalent among schizophrenic patients than the general population. Electronic cigarettes are becoming increasingly popular with smokers worldwide. To date there are no large randomized trials of electronic cigarettes in schizophrenic smokers. A well-designed trial is needed to compare efficacy and safety of these products in this special population.

**Methods/Design:**

Intervention: We have designed a randomized controlled trial investigating the efficacy and safety of electronic cigarette. The trial will take the form of a prospective 12-month randomized clinical study to evaluate smoking reduction, smoking abstinence and adverse events in schizophrenic smokers not intending to quit. We will also monitor quality of life, neurocognitive functioning and measure participants’ perception and satisfaction of the product.

Outcome measures: A ≥50% reduction in the number of cigarettes/day from baseline, will be calculated at each study visit (“reducers”). Abstinence from smoking will be calculated at each study visit (“quitters”). Smokers who leave the study protocol before its completion and will carry out the Early Termination Visit or who will not satisfy the criteria of “reducers” and “quitters” will be defined “non responders”.

Statistical analysis: The differences of continuous variables between the three groups will be evaluated with the Kruskal-Wallis Test, followed by the Dunn multiple comparison test. The differences between the three groups for normally distributed data will be evaluated with ANOVA test one way, followed by the Newman-Keuls multiple comparison test. The normality of the distribution will be evaluated with the Kolmogorov-Smirnov test. Any correlations between the variables under evaluation will be assessed by Spearman r correlation. To compare qualitative data will be used the Chi-square test.

**Discussion:**

The main strengths of the SCARIS study are the following: it’s the first large RCT on schizophrenic patient, involving in and outpatient, evaluating the effect of a three-arm study design, and a long term of follow-up (52-weeks).

The goal is to propose an effective intervention to reduce the risk of tobacco smoking, as a complementary tool to treat tobacco addiction in schizophrenia.

**Trial registration:**

ClinicalTrials.gov, NCT01979796.

## Background

It is well established in studies across several countries that tobacco smoking is more prevalent among schizophrenic patients than the general population [1]. For example, in the US, 80% or more of schizophrenic patients smoke, compared to approximately 20% of the general population [2]. An increased rate of smoking among subjects with schizophrenia contributes to multiple negative health effects, including higher rates of coronary heart disease, hypertension, respiratory disease and lung cancer than in the general population [3]. Randomized clinical trials of currently marketed smoking cessation products (varenicline, bupropion, nicotine replacement therapy) in schizophrenia have shown limited effects [4]. This scenario is further complicated by the belief that quitting smoking will worsen psychiatric symptoms, or that these patients have little or no interest in quitting. Moreover, the prescribing information for bupropion and varenicline, two important first-line medications for nicotine dependence, carry a *black-box* warning highlighting an increased risk of psychiatric symptoms and suicidal ideation in patients reporting any history of psychiatric illness [5].

Electronic cigarettes (e-cigarettes) are becoming increasingly popular with smokers worldwide. Users report buying them to help quit smoking, to reduce cigarette consumption, to relieve tobacco withdrawal symptoms, and to continue having a smoking experience, but with reduced health risks [6]. A recent randomized controlled trial (RCT), showed that smokers not immediately willing to quit who used e-cigarettes substantially decreased daily cigarette consumption without significant side effects [7]. In a prospective 12-month pilot study, e-cigarettes were shown to substantially decrease cigarette consumption without causing significant side effects in schizophrenic smokers not intending to quit [8], however, in a recent large randomized clinical trial of e-cigarettes conducted in 300 smokers, side effects that are commonly recorded during smoking cessation trials using drugs for nicotine dependence were infrequently reported during the course of the study [7]; for example, at week-2, hunger, insomnia, irritability, anxiety, and depression were reported by 6.5%, 4.0%, 3.5%, 3.0% and 2.0% of participants, respectively. Moreover, no serious adverse events (AEs) (that is, major depression, abnormal behavior or any event requiring an unscheduled visit to the family practitioner or hospitalization) occurred during the study. Quitters also reported improved quality of life, which could be used to motivate attempts to quit by individuals with concerns about what life will be like without cigarettes [9] Some authors suggest the hypothesis that smoking causes cognitive decline and loss of gray-matter tissue in the brain over time [10].

### Research plan

To date there are no large randomized trials of e-cigarettes in schizophrenic smokers. A well-designed trial is needed to compare efficacy and safety of these products in this special population.

### Objectives

#### Primary

The primary outcome is the evaluation of the efficacy and safety of a popular electronic cigarette brand. The trial will take the form of a prospective 12-month randomized clinical study to evaluate smoking reduction, smoking abstinence and AEs in schizophrenic smokers not intending to quit, experimenting with two different nicotine strengths of e-cigarette.

#### Secondary outcomes

The secondary outcomes are to monitor AEs, quality of life, neurocognitive functioning, and spirometry, and to measure participants’ perception of and satisfaction with the product.

## Methods

### Participants

One hundred fifty-three schizophrenic regular smokers will be recruited in three centers (Smoking Cessation Centre University of Catania Medical Hospital; Psychiatric Unit of University of Catania Medical Hospital; CTA “Villa Chiara” Psychiatric Clinic) following placement of advertisements in local newspapers and radio/television network inviting them to try the e-cigarette to reduce the risk of tobacco smoking. Each participating center will include 51 eligible smokers in the study. The study protocol has been approved by the Ethical Committee of the University of Catania (Record number 674; 30-05-2013) in accordance with the Helsinki Declaration. Written informed consent will be obtained by all participants.

Inclusion criteria are: 1) schizophrenic subjects (according to DSM-IV-TR criteria) from Sicily (Italy), who smoke tobacco cigarettes, in who are in the stable phase of illness (no relapse/hospitalization and/or need to change psychopharmacological treatment in the last 12 months); 2) smoked ≥10 factory-made cigarettes/day for at least the past five years; 3) age 18 to 65 years; 4) in good general health (absence of cancer, acute myocardial infarction, unstable angina, severe cardiac arrhythmia, recent cerebrovascular incident, or severe atherosclerosis); 5) not currently attempting to quit smoking or wishing to do so in the next (a specific test will be included to check their unwillingness to quit) 6 months; 6) committed to follow the trial procedures.

Exclusion criteria are: 1) use of smokeless tobacco or nicotine replacement therapy; 2) pregnancy or breastfeeding; 3) current or recent (less than 1 year) history of alcohol and/or drug abuse; 4) other significant comorbidities according to the Investigator’s clinical assessment (for example, cancer, acute myocardial infarction, unstable angina, severe cardiac arrhythmia, recent cerebrovascular incident, or severe atherosclerosis.

### Products tested: electronic cigarette

**Figure 1:**
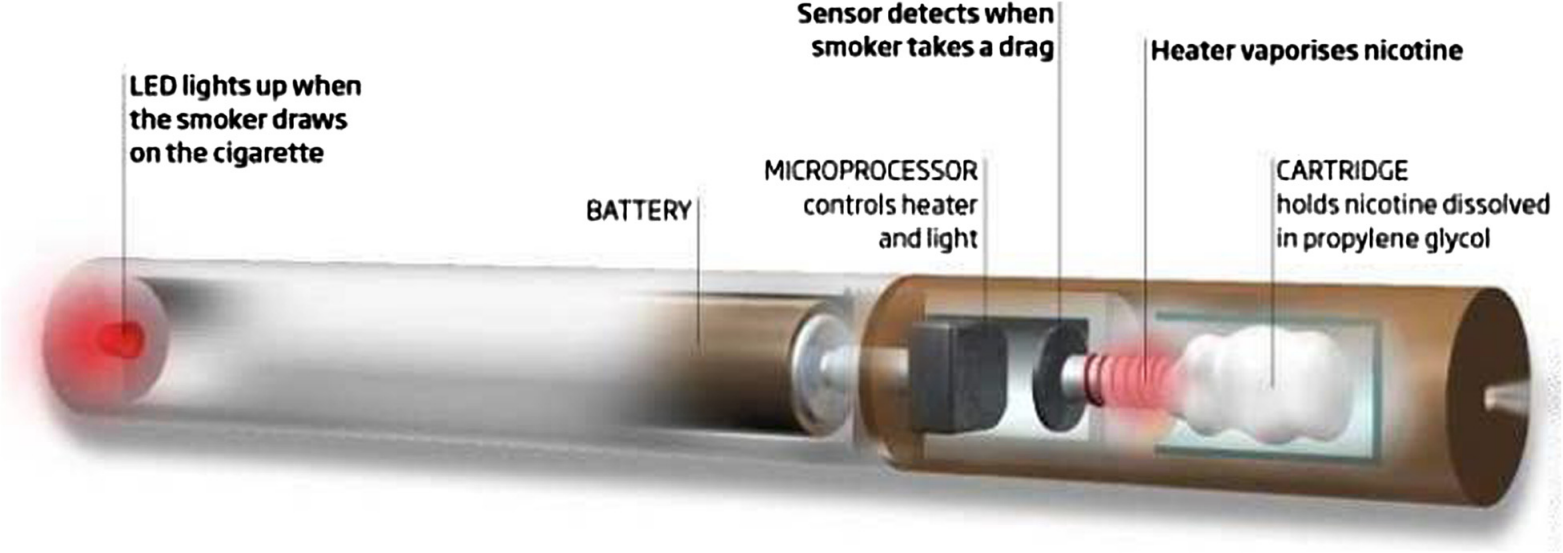
**Electronic cigarette.** It is a disposable model that closely resembles the shape of a tobacco cigarette, with a rechargeable 3.7-V, 120mAh, lithium-ion battery. The electronic cigarette contains a liquid solution of vegetable glycerin in which nicotine or an aroma is dissolved.

The e-cigarette will be used in this study. It is a disposable model that closely resembles the shape of a tobacco cigarette. Its heating element in the atomizer is activated by a rechargeable 3.7-V, 120mAh, lithium-ion battery. Standard performance guarantees the equivalent of 300 to 400 puffs/each disposable product. The e-cigarette contains a liquid solution of vegetable glycerin in which nicotine or an aroma is dissolved. Two different types of disposable e-cigarette will be provided for the study; high-nicotine (with 24 mg nicotine) and no-nicotine (with a tobacco aroma). Detailed toxicology and nicotine content analyses of the e-cigarette has been provided to the local ethics review boards (ERBs) (Figure 1).

### Products tested: the PAIPO nicotine-free inhalator (EchosSrl, Milan, Italy)

**Figure 2:**
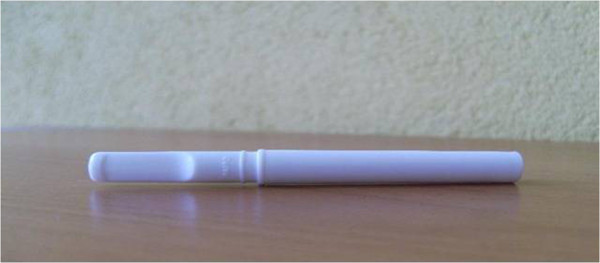
**The PAIPO nicotine-free inhalator (EchosSrl, Milan, Italy).** It is a plastic device that resembles a cigarette and is intended to replace some of the rituals associated with smoking gestures. The plastic device contains a sponge filter soaked in natural oil enriched with extracts of different aromas.

This plastic device resembles a cigarette and is intended to replace some of the rituals associated with smoking gestures, for example, the hand-to-mouth action of smoking, and handling and manipulation of cigarettes. The plastic device contains a sponge filter soaked in natural oil enriched with extracts of different aromas. For this study protocol we will provide a placebo grapefruit-flavor PAIPO inhalator (Figure 2).

### Study design

The study is a three-arm, randomized controlled, clinical trial designed to assess the efficacy and safety of the e-cigarette with 24 mg nicotine, the e-cigarette with no nicotine, and the PAIPO nicotine-free inhalator. We adhered to Consolidated Standards of Reporting Trials (CONSORT) guidelines in the design of the trial.

At screening the diagnosis of schizophrenia will be determined using the structured clinical interview for DSM-IV axis I disorders - clinical version (SCID-I-CV); subjects with organic psychosis, learning disabilities or requiring a translator because of lack of Italian fluency will be excluded from the study. The psychopathological status of the participants will be assessed by a trained psychiatrist (blinded to the treatment conditions) using the positive and negative syndrome scale (PANSS) to evaluate the severity of illness. Written informed consent will be obtained by all eligible participants at this visit. Screening in brief is as follows: informed consent; inclusion/exclusion criteria; Case Report Form”CRF (socio-demographic factors; smoking history; SCID-I-CV; PANSS); if eligible book next appointment in 1 week.

**Figure 3:**
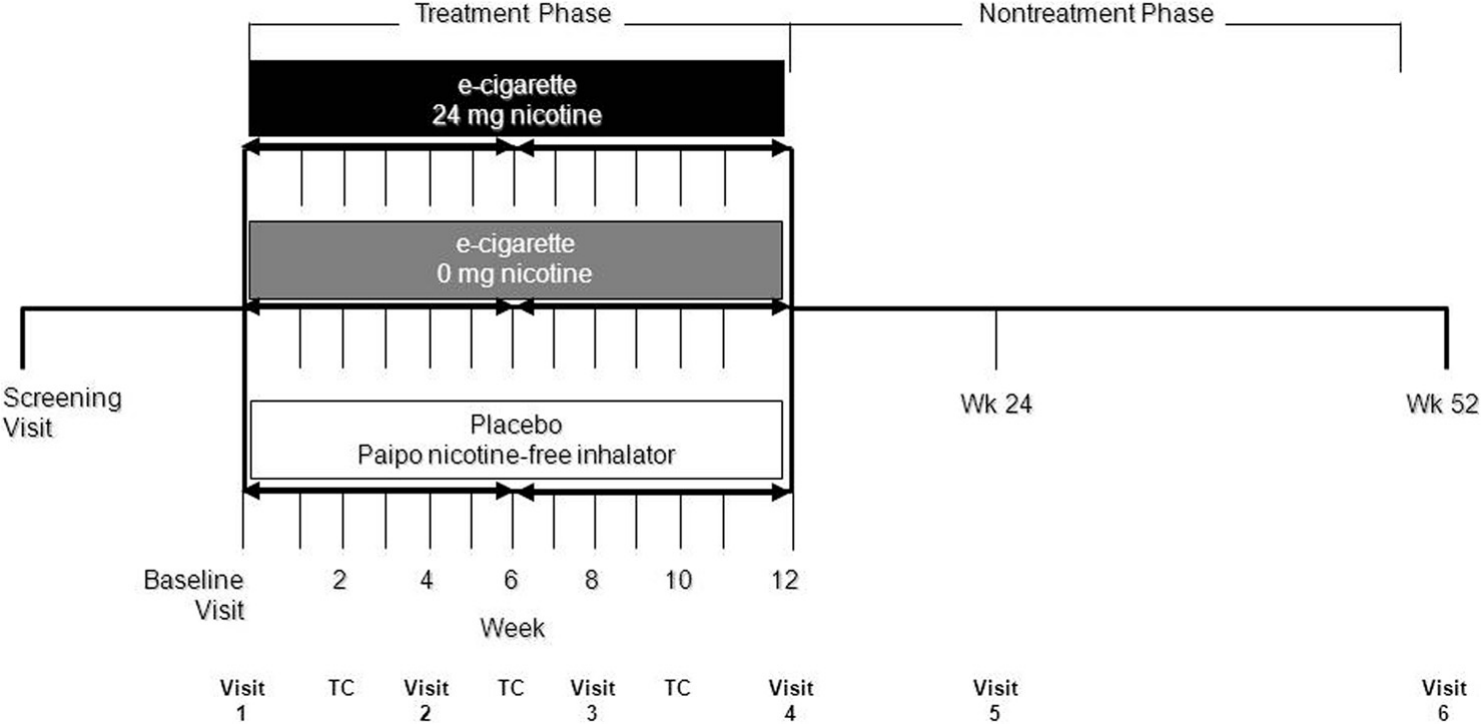
**Study design.** The study is a three-arm, randomized controlled, clinical trial designed to assess the efficacy and safety of the e-cigarette with 24 mg nicotine, the e-cigarette with no (0 mg) nicotine and the PAIPO nicotine-free inhalator.

At baseline (BL), participants will be randomized into three separate study groups. The randomization sequence will be computer generated by permuted blocks of five for each of the three study conditions (A, B, and C). Participants randomized in study group A will receive a 12-week supply of the e-cigarette with 24 mg of nicotine; those in study group B, a 12-week supply of the e-cigarette 0 mg of nicotine; participants in study group C will receive a 12-week supply of the PAIPO nicotine-free inhalator. A prospective evaluation of efficacy and safety will be repeated at an additional follow-up visit at 24 weeks. The study will consist of a total of seven visits: a screening visit, a BL visit and five follow-up visits (at weeks 4, 8, 12, 24, and 52). Three telephone contacts (TC) will be scheduled during the treatment phase (at weeks 2, 6, and 10) (Figure 3).

### Study schedule

Participants will attend their study visits at the smoking cessation clinic at approximately the same time of day. With the exception of the BL study day, most visits will take approximately 10 to 15 minutes to complete. The BL visit will be carried out within seven days of the screening visit. At BL (study visit 1), socio-demographic factors, and detailed smoking history will be annotated and individual pack-years (pack/yrs) calculated, together with the ratings of quality of life and neurocognitive functioning assessed by the quality of life scale (QLS) and brief assessment of cognition in schizophrenia (BACS), respectively. Physical cigarette dependence and behavioral dependence will be measured with the Fagerstrom test for cigarette dependence (FTCD) and Glover-Nilsson smoking behavioral questionnaire (GN-SBQ), respectively. Additionally, levels of carbon monoxide in exhaled breath (eCO) will be measured using a portable device (Micro CO, Micro Medical Ltd, Kent, UK). Vital signs (heart rate (HR) and blood pressure (BP)), body weight, spirometry, and AEs will also be recorded at baseline.

Participants will be given a free supply of e-cigarettes or PAIPO inhalators (on average, a 4-week supply of e-cigarettes) and instructed on how to correctly use them. Key trouble-shooting support will be provided and phone numbers will be supplied for medical assistance. A full 4-week supply of nicotine or no-nicotine e-cigarettes, or PAIPO inhalators (depending on the study-arm allocation) will be also provided so that every participant will have enough disposable products for each study period; a study diary recording will be used to check for the most common AEs related to the use of e-cigarettes (for example, dry cough, mouth irritation, throat irritation, headache, shortness of breath, nausea et cetera). No emphasis on encouragement, motivation and reward for the smoking cessation effort will be provided, because this study was intended to monitor schizophrenic smokers who are unwilling to quit. Participants will be instructed to return every four weeks to obtain their 4-weekly supply of e-cigarettes or PAIPO inhalators.

The BL (visit 1) in brief is as follows: CRF (physical examination, FTCD, GN-SBQ, QLS, BACS, HR, BP, weight, eCO, spirometry, AEs); craving/visual analog scale (VAS); MNWS; randomization into either study group A (e-cigarette 24 mg), B (e-cigarette 0 mg), or C (PAIPO); dispense 4-weeks supply of nicotine e-cigarette, no-nicotine e-cigarette or PAIPO inhalator (depending on the study-arm allocation); dispense 4-week study diary for AEs; book for next appointment in 4 weeks (week 4; study visit 2).

By and large, at week 4 (study visit 2), and week 8 (study visit 3), participants will a) receive further study diaries for the residual study periods; b) have their eCO levels and vital signs recorded; and c) return their completed study diaries and used study products. At the end of the week-12 study visit, no more products will be provided by the investigators.

Week-4 (visit 2) in brief is as follows: collect 4-week study diary for AEs; CRF (number of cigarettes/day, eCO, HR, BP); craving/VAS; MNWS; PANSS; smokers’ product preference survey; dispense next 4-week supply of nicotine e-cigarette, no-nicotine e-cigarette or PAIPO inhalator (depending on the study-arm allocation); dispense next 4-week study diary for AEs; book for next appointment in 4 weeks (week 8; study visit 3).

Week-8 and -12 (visits 3 and 4) in brief are as follows: collect 4-week study diary for AEs; CRF (number of cigarettes/day, eCO, HR, BP); craving/VAS; MNWS; PANSS; smokers’ product preference survey; dispense next 4-week supply of nicotine e-cigarette, no-nicotine e-cigarette or PAIPO inhalator (depending on the study-arm allocation); book for next appointment in 12 weeks (week24; study visit 5).

Week 24 (visit 5) in brief is as follows: collect 4-week study diary for AEs; CRF (number of cigarettes/day, eCO, HR, BP); craving/VAS; MNWS; PANSS; BACS; QLS; smokers’ product preference survey; spirometry; book for next appointment for the final visit (week 52; study visit 6).

Week 52 (visit 6) in brief is as follows: CRF (number of cigarettes/day, eCO, HR, BP); craving/VAS; MNWS; PANSS; BACS; QLS; smokers’ product preference survey; spirometry.

Study participants will attend a final follow-up visit at week 52 to report product use and the number of any tobacco cigarettes smoked (from which reduction and quit rates could be calculated), and to re-check eCO levels. Quality of life, neurocognitive functioning and psychopathological status will be assessed by QLS, BACS, and PANSS, respectively. Vital signs (HR and BP), body weight, and spirometry will be recorded again as well as participants’ perceptions of the specific product they are using [11].

Early termination visit (unscheduled visit) is as follows: CRF (number of cigarettes/day, eCO, HR, BP); craving/VAS; MNWS; PANSS; BACS; QLS; smokers’ product preference survey; spirometry (Table 1).

**Table 1:** Study schedule

**Procedure**		**BL Visit**	**Wk4**	**Wk8**	**Wk12**	**Wk24**	**Wk52**	**Early termination visit**
		**Visit 1**	**Visit 2**	**Visit 3**	**Visit 4**	**Visit 5**	**Visit 6**	
Informed consent		**X**						
Inclusion/exclusion criteria (review)		**X**						
**CRF**	Sociodemografic factors	**X**						
	Smoking Hx	**X**						
	no. cig/day	**X**	**X**	**X**	**X**	**X**	**X**	**X**
	Physical examination	**X**					**X**	
	FTDC, GN-SBQ, QLS, BACS	**X**			**X**	**X**	**X**	**X**
	eCO	**X**	**X**	**X**	**X**	**X**	**X**	**X**
	HR, BP	**X**	**X**	**X**	**X**	**X**	**X**	**X**
	Weight	**X**				**X**	**X**	
	AEs	**X**	**X**	**X**	**X**	**X**	**X**	**X**
Randomization into either study group A (Ecig 24 mg), B (Ecig o mg), or C (paipo)		**X**						
Dispense 4-weeks supply of either nicotine or no-nicotine or paipo (depending on arm allocation)		**X**	**X**	**X**	**X (x3)**			
Dispense 4-weeks’ study diary for AEs		**X**	**X**	**X**	**X (x3)**			
Spirometry		**X**			**X**	**X**	**X**	**X**
Book for next appointment in 4 weeks (week-4; study visit 2)		**X**	**X**	**X**	**X (x3)**	**X(x6)**		
Craving/VAS		**X**	**X**	**X**	**X**	**X**	**X**	**X**
MNWS		**X**	**X**	**X**	**X**	**X**	**X**	**X**
PANSS			**X**	**X**	**X**	**X**	**X**	**X**

### Study outcome measures

A reduction ≥50% in the number of cigarettes/day from BL, defined as self-reported reduction in the number of cigarettes/day (≥50%) compared to BL (together with an eCO levels reduction, to objectively document a reduction from baseline), will be calculated at each study visit (referred to as reducers). Abstinence from smoking, defined as complete self-reported abstinence from tobacco smoking - not even a puff (together with an eCO concentration ≤7 ppm), will be calculated at each study visit (referred to as quitters). Smokers who do not satisfy the criteria of reducers or quitters will be defined non responders. Smokers who leave the study protocol before its completion due to lack of efficacy or poor tolerability of the product under investigation, will carry out the early termination visit and will be defined as non responders.

AEs, symptoms thought to be related to tobacco smoking and e-cigarette use, and to withdrawal from nicotine, will be annotated at BL and at each subsequent study visit on the AE page of the study diary. Vital signs will be also recorded. The number and the percentage of subjects experiencing AEs, adverse reactions, serious AEs and AEs leading to study withdrawal will be summarized by treatment group. AEs will also be summarized by system organ class and preferred term using the *MedDRA* dictionary.

Quality of life, neurocognitive functioning and psychopathological status will be reassessed by QLS, BACS and PANSS, respectively. Participants’ perceptions and liking of the product will be assessed by asking them to rate their levels of satisfaction with the products compared to their own cigarettes using a VAS, scoring from 0 to 10 points (0 = completely unsatisfied, 10 = fully satisfied). Using the same scale, they will also rate how much they miss their own brand (0 = did not miss it at all, 10 = missed it too much) and whether they would recommend it to a friend/relative (0 = not recommended at all, 10 = absolutely recommended).

### Sample size

All statistical tests were two-tailed and were considered to be statistically significant at *P* < 0.05. A sample size of 133 subjects was calculated accepting the following parameters: effect size medium = 0.30; alpha = 0.05; 1-ß = 0.80. Considering a drop-out rate of 15%, a total sample size of 153 smokers was determined. The smokers will be randomized into the three arms of our study protocol (51 smokers for each arm).

### Statistical methods

Statistical analysis will be performed with SPSS 20.0 (Statistical Package for Social Sciences Program, IBM). Continuous variables will be described with mean, standard deviation, median, minimum, maximum and the 25th and 75th percentiles. Categorical variables will be described with percentages and absolute frequencies. The differences in continuous variables between the three groups will be evaluated with the Kruskal-Wallis test, followed by the Dunn multiple comparison test. The differences between the three groups for normally distributed data will be evaluated with one-way analysis of variance (ANOVA), followed by the Newman-Keuls multiple comparison test. The normality of the distribution will be evaluated with the Kolmogorov-Smirnov test. Any correlation between the variables under evaluation will be assessed by Spearman *r* correlation. To compare qualitative data we will use the Chi-square test with the Yates correction or the Fisher exact test.

## Discussion

People with schizophrenia have more than five times the odds of current smoking and smoking cessation rates are much lower than in the general population [12]. In addition, smokers with schizophrenia smoke more heavily and extract more nicotine from each cigarette [13]. Even patients with first-episode psychosis tend to have high prevalence of tobacco use and are much more likely to smoke than age-matched controls, as confirmed in a recent meta-analysis (odds ratio = 6.04) [14].

Chronic cigarette smoking has been suggested as a major contributing factor to higher morbidity and mortality in schizophrenic patients, especially in people aged 35 to 54 years [15]. In addition to such adverse health effects, cigarette smoking clearly represents a huge financial burden on patients with schizophrenia. Money spent on cigarettes is not being spent on clothing, leisure pursuits and personal possessions, which could help to increase the quality of life of these patients [16].

Through their smoking habits people with schizophrenia are contributing substantially to the cost of their own care (those who smoke return 18 to 31% of their benefits to the Treasury) [17]. However, heavy smoking in schizophrenia cannot simply be viewed as a bad habit. Rather, self-medication of clinical symptoms and side effects of antipsychotic drugs appear to play a major role [18]. The cognitive approach to the self-medication hypothesis maintains that patients smoke in an attempt to improve their cognitive deficits. Nicotine improves deficits in attention and working memory in schizophrenic patients even in first episode [19-22].

Healthcare providers previously have not offered tobacco dependence treatment to patients with schizophrenia, probably secondarily to stigma or lack of information [23]. Moreover, smokers with schizophrenia have a more severe nicotine dependence compared to smokers without schizophrenia [14]. Hence, interventions may not be as effective as they have been shown to be in the general population. We also need to consider the safety of these interventions, particularly in relation to drug therapy. In addition, nicotine withdrawal can cause symptoms such as depression, anxiety and irritability. All these factors may contribute to changes in the mental state of these patients, and the extent of these changes remains unclear [24]. For these reasons, recent initiatives have aimed to study smoking habits and specific intervention programs for smokers with schizophrenia [7,8,15,24].

The proposed randomized trial consists of an investigation of the efficacy and safety of e-cigarettes in schizophrenic smokers. To date there are no large randomized trials of e-cigarettes in schizophrenic smokers. The main strengths of the SCARIS study are the following: it is the first large RCT on schizophrenic patients, involving inpatients and outpatients, with a three-arm study design and long-term follow up (52 weeks).

The goal is to propose an effective intervention to reduce the risk of tobacco smoking, as a complementary tool to treat smokers with schizophrenia. Therefore, as our clinical experience in the smoking cessation/reduction treatment of smokers suffering of schizophrenia evolves, the smoking cessation and reduction in schizophrenia (SCARIS) study protocol stands as a unique milestone robust trial, which will contribute to our fundamental understanding of the role of the e-cigarette in smoking cessation/reduction and its influence on the health status of this particular population.

## Trial status

We expect to start recruiting participants in September 2014.

## Abbreviations

BACS: brief assessment of cognition in schizophrenia; BL: baseline; BP: blood pressure; eCO: carbon monoxide in exhaled breath; e-cigarettes: electronic cigarettes; FTCD: Fagerstrom test for cigarette dependence; GN-SBQ: Glover-Nilsson smoking behavioral questionnaire; HR: heart rate; PANSS: positive and negative syndrome scale; QLS: quality of life scale; RCT: randomized controlled trial; SCARIS: smoking cessation and reduction in schizophrenia; SCID-I-CV: structured clinical interview for DSM-IV axis I disorders - clinical version; VAS: visual analog scale.

## Competing interests

RP has received lecture fees and research funding from Pfizer and GlaxoSmithKline, manufacturers of stop smoking medications. He has served as a consultant for Pfizer and Arbi Group Srl, the distributor of the Categoria™ e-cigarette. RP currently serves as Chief Scientific Advisor for the Italian Anti-Smoking League (LIAF). The authors have no other competing interests to declare.

## Authors’ contributions

PC and GM were responsible for the conception and design of the study and wrote the first drafts of the study protocol. RP, RA MS, MM,AA, FP and EA conceived and participated in its design and coordination and helped to draft the manuscript. FP performed statistical analysis. All authors read and approved the final manuscript.
